# Ethyl 5-cyano-4-[2-(2,4-dichloro­phen­oxy)acetamido]-1-phenyl-1*H*-pyrrole-3-carboxyl­ate

**DOI:** 10.1107/S1600536809027809

**Published:** 2009-07-22

**Authors:** Jing Xu, Hui Sheng, Mei He, Ping He

**Affiliations:** a Institute of Medicinal Chemistry, Yunyang Medical College, Shiyan 442000, People’s Republic of China; b School of Basic Medical Science, Yunyang Medical College and Yunyang Medical College Library, Shiyan 442000, People’s Republic of China; c Department of Pharmacy, Affiliated Renmin Hospital, Yunyang Medical College, Shiyan 442000, People’s Republic of China; d Key Laboratory of Pesticides & Chemical Biology, Ministry of Education, Central China Normal University, Wuhan 430079, People’s Republic of China

## Abstract

In the title compound, C_22_H_17_Cl_2_N_3_O_4_, the pyrrole ring and the 2,4-dichloro­phenyl group form a dihedral angle of 8.14 (13)°; the phenyl ring is twisted with respect to the pyrrole ring, forming a dihedral angle of 60.77 (14)°. The C=O bond length is 1.213 (3) Å, indicating that the mol­ecule is in the keto form, associated with a –CONH– group, and the amide group adopts the usual *trans* conformation. The mol­ecule is stabilized by an intra­molecular N—H⋯O hydrogen-bonding inter­action. In the crystal, the stacked mol­ecules exhibit inter­molecular C—H⋯O and C—H⋯N hydrogen-bonding inter­actions.

## Related literature

For the preparation and biological activity of acid amides, see: Xue *et al.* (2007 ▶); Li *et al.* (1995 ▶). For related structures, see: He *et al.* (2007*a* ▶,*b* ▶).
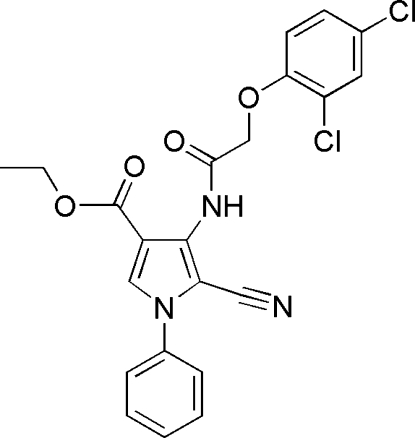


## Experimental

### 

#### Crystal data

C_22_H_17_Cl_2_N_3_O_4_
*M* *_r_* = 458.29
Monoclinic, 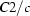

*a* = 32.8846 (18) Å
*b* = 7.6224 (4) Å
*c* = 17.2834 (9) Å
γ = 89.773 (1)°
*V* = 4332.2 (4) Å^3^
*Z* = 8
Mo *K*α radiation
μ = 0.33 mm^−1^
*T* = 298 K
0.10 × 0.10 × 0.10 mm

#### Data collection

Bruker SMART 4K CCD area-detector diffractometer
Absorption correction: multi-scan (*SADABS*; Sheldrick, 2003 ▶) *T*_min_ = 0.968, *T*_max_ = 0.968
16521 measured reflections
4279 independent reflections
3034 reflections with *I* > 2σ(*I*)
*R*_int_ = 0.081

#### Refinement

*R*[*F* ^2^ > 2σ(*F* ^2^)] = 0.066
*wR*(*F* ^2^) = 0.154
*S* = 0.99
4279 reflections
285 parameters
H atoms treated by a mixture of independent and constrained refinement
Δρ_max_ = 0.32 e Å^−3^
Δρ_min_ = −0.31 e Å^−3^

### 

Data collection: *SMART* (Bruker, 2001 ▶); cell refinement: *SAINT-Plus* (Bruker, 2001 ▶); data reduction: *SAINT-Plus*; program(s) used to solve structure: *SHELXS97* (Sheldrick, 2008 ▶); program(s) used to refine structure: *SHELXL97* (Sheldrick, 2008 ▶); molecular graphics: *PLATON* (Spek, 2009 ▶); software used to prepare material for publication: *SHELXTL* (Sheldrick, 2008 ▶).

## Supplementary Material

Crystal structure: contains datablocks I, global. DOI: 10.1107/S1600536809027809/at2840sup1.cif

Structure factors: contains datablocks I. DOI: 10.1107/S1600536809027809/at2840Isup2.hkl

Additional supplementary materials:  crystallographic information; 3D view; checkCIF report

## Figures and Tables

**Table 1:** Hydrogen-bond geometry (Å, °)

*D*—H⋯*A*	*D*—H	H⋯*A*	*D*⋯*A*	*D*—H⋯*A*
N1—H1⋯O3	0.82 (2)	2.15 (3)	2.813 (3)	137 (2)
C7—H7*A*⋯O2^i^	0.97	2.57	3.341 (3)	137
C3—H3⋯N3^i^	0.93	2.61	3.312 (3)	133

Symmetry code: (i) 
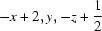
.

**Fig. 1.**
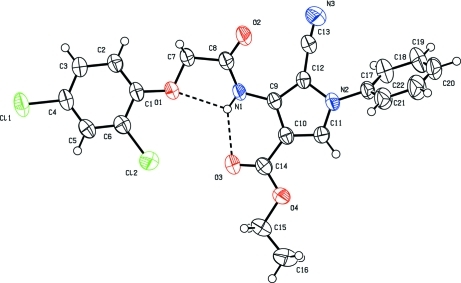
A view of the molecule of (I) showing the atom-labeling scheme. Displacement ellipsoids are drawn at the 50% probability level. Intramolecular hydrogen bonds are drawn as a dashed line.

**Fig. 2.**
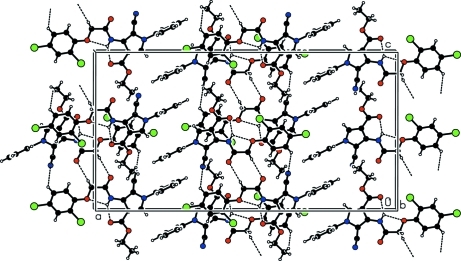
Part of the crystal structure of (I), showing hydrogen bonds stacking interactions.

## supplementary crystallographic information

### Comment

The chemical and pharmacological properties of acid amides have been investigated extensively, owing to their potentially beneficial chemical and biological activties (Xue *et al.*, 2007 and Li *et al.*, 1995). As part of our studies on the synthesis and characterization of related compounds (He *et al.*, 2007a,b), we report here the synthesis and crystal structure of ethyl 4-(2-(2,4-dichlorophenoxy)acetamido)-5-cyano-1-phenyl-1*H*-pyrrole- 3-carboxylate, (I).

Within the molecule of (I), the bond lengths and angles present no unusual features. The pyrrole ring and the 2,4-dichlorophenyl group form a dihedral angle of 8.14 (13)°, the C17—C22 phenyl ring is twisted with respect to pyrrole ring, with a dihedral angle of 60.77 (14)°. The C=O bond length is 1.213 (3) Å, indicating that the molecule is in the keto form (Fig. 1), associated with –CONH– moiety, and the amide group adopts the usual *trans* conformation. The crystal structure is stabilized by intramolecular N—H···O hydrogen bonds interactions. In additional, the stacked molecules exhibit intermolecular C—H···O and C—H···N hydrogen bonds interactions (Fig 2. Table 1).

### Experimental

To a solution of the ethyl 4-amino-5-cyano-1-phenyl-1*H*-pyrrole-3-carboxylate (3 mmol) in dry dichloromethane (15 ml) was added 2-(2,4-dichlorophenoxy)acetyl chloride at 273–278 K. After stirring the reaction mixture for 4 h from 273–278 K to room temperature. The solution was concentrated under reduced pressure and the residue was recrystallized from ethanol to give the title compound in a yield of 45%. Crystals suitable for single-crystal X-ray diffraction were obtained by recrystallization from a mixed solvent of ethanol and dichloromethane (1:3 *v*/*v*) at room temperature.

### Refinement

The carbon-bound hydrogen atoms were positioned geometrically and refined using a riding model with C—H = 0.93 Å, *U*_iso_=1.2*U*_eq_ (C) for C*sp*^2^, C—H = 0.97 Å, *U*_iso_ = 1.2*U*_eq_ (C) for CH_2_ and C—H = 0.96 Å, *U*_iso_ = 1.5*U*_eq_ (C) for CH_3_. The H atom of the NH group was found from a difference Fourier map and refined with a fixed *U*_iso_ of 0.05.

### Crystal data

**Table:**

C_22_H_17_Cl_2_N_3_O_4_	*F*(000) = 1888
*M**_r_* = 458.29	*D*_x_ = 1.401 Mg m^−^^3^
Monoclinic, *C*2/*c*	Mo *K*α radiation, λ = 0.71073 Å
Hall symbol: -C 2yc	Cell parameters from 4400 reflections
*a* = 32.8846 (18) Å	θ = 2.5–26.3°
*b* = 7.6224 (4) Å	µ = 0.33 mm^−^^1^
*c* = 17.2834 (9) Å	*T* = 298 K
β = 90°	Block, yellow
*V* = 4332.2 (4) Å^3^	0.10 × 0.10 × 0.10 mm
*Z* = 8	

### Data collection

**Table:**

Bruker SMART 4K CCD area-detector diffractometer	4279 independent reflections
Radiation source: fine-focus sealed tube	3034 reflections with *I* > 2σ(*I*)
graphite	*R*_int_ = 0.081
φ and ω scans	θ_max_ = 26.0°, θ_min_ = 2.4°
Absorption correction: multi-scan (*SADABS*; Sheldrick, 2003)	*h* = −40→39
*T*_min_ = 0.968, *T*_max_ = 0.968	*k* = −9→9
16521 measured reflections	*l* = −21→21

### Refinement

**Table:**

Refinement on *F*^2^	Secondary atom site location: difference Fourier map
Least-squares matrix: full	Hydrogen site location: inferred from neighbouring sites
*R*[*F*^2^ > 2σ(*F*^2^)] = 0.066	H atoms treated by a mixture of independent and constrained refinement
*wR*(*F*^2^) = 0.154	*w* = 1/[σ^2^(*F*_o_^2^) + (0.055*P*)^2^] where *P* = (*F*_o_^2^ + 2*F*_c_^2^)/3
*S* = 0.99	(Δ/σ)_max_ = 0.001
4279 reflections	Δρ_max_ = 0.32 e Å^−^^3^
285 parameters	Δρ_min_ = −0.31 e Å^−^^3^
0 restraints	Extinction correction: *SHELXL97* (Sheldrick, 2008), Fc^*^=kFc[1+0.001xFc^2^λ^3^/sin(2θ)]^-1/4^
Primary atom site location: structure-invariant direct methods	Extinction coefficient: 0.0042 (4)

### Special details

**Table:**

Geometry. All e.s.d.'s (except the e.s.d. in the dihedral angle between two l.s. planes) are estimated using the full covariance matrix. The cell e.s.d.'s are taken into account individually in the estimation of e.s.d.'s in distances, angles and torsion angles; correlations between e.s.d.'s in cell parameters are only used when they are defined by crystal symmetry. An approximate (isotropic) treatment of cell e.s.d.'s is used for estimating e.s.d.'s involving l.s. planes.
Refinement. Refinement of *F*^2^ against ALL reflections. The weighted *R*-factor *wR* and goodness of fit *S* are based on *F*^2^, conventional *R*-factors *R* are based on *F*, with *F* set to zero for negative *F*^2^. The threshold expression of *F*^2^ > σ(*F*^2^) is used only for calculating *R*-factors(gt) *etc*. and is not relevant to the choice of reflections for refinement. *R*-factors based on *F*^2^ are statistically about twice as large as those based on *F*, and *R*- factors based on ALL data will be even larger.

### Fractional atomic coordinates and isotropic or equivalent isotropic displacement parameters (Å^2^)

**Table:**

	*x*	*y*	*z*	*U*_iso_*/*U*_eq_	
C1	1.06329 (6)	0.1879 (3)	0.05715 (13)	0.0426 (6)	
C2	1.09068 (7)	0.1312 (3)	0.11185 (14)	0.0466 (6)	
H2	1.0813	0.0801	0.1573	0.056*	
C3	1.13204 (7)	0.1495 (3)	0.09995 (14)	0.0472 (6)	
H3	1.1505	0.1107	0.1369	0.057*	
C4	1.14550 (7)	0.2257 (3)	0.03284 (15)	0.0463 (6)	
C5	1.11863 (7)	0.2840 (3)	−0.02255 (14)	0.0470 (6)	
H5	1.1282	0.3351	−0.0679	0.056*	
C6	1.07783 (7)	0.2659 (3)	−0.01021 (14)	0.0469 (6)	
C7	1.00695 (6)	0.1112 (4)	0.13490 (13)	0.0558 (7)	
H7A	1.0184	0.1792	0.1770	0.067*	
H7B	1.0147	−0.0103	0.1420	0.067*	
C8	0.96114 (7)	0.1270 (4)	0.13558 (14)	0.0511 (7)	
C9	0.90125 (6)	0.1809 (3)	0.05703 (13)	0.0368 (5)	
C10	0.88509 (6)	0.2629 (3)	−0.00962 (13)	0.0399 (6)	
C11	0.84392 (7)	0.2760 (3)	0.00079 (14)	0.0440 (6)	
H11	0.8259	0.3266	−0.0340	0.053*	
C12	0.86902 (6)	0.1425 (3)	0.10524 (12)	0.0387 (5)	
C13	0.86531 (6)	0.0454 (3)	0.17456 (14)	0.0446 (6)	
C14	0.90849 (7)	0.3115 (3)	−0.07786 (13)	0.0431 (6)	
C15	0.90645 (8)	0.4263 (4)	−0.20453 (14)	0.0589 (7)	
H15A	0.9189	0.3226	−0.2267	0.071*	
H15B	0.9277	0.5115	−0.1943	0.071*	
C16	0.87619 (9)	0.5007 (4)	−0.25914 (15)	0.0693 (8)	
H16A	0.8568	0.4119	−0.2729	0.104*	
H16B	0.8898	0.5412	−0.3048	0.104*	
H16C	0.8624	0.5970	−0.2350	0.104*	
C17	0.79429 (7)	0.2161 (4)	0.10469 (13)	0.0448 (6)	
C18	0.77391 (8)	0.0684 (4)	0.12715 (16)	0.0647 (8)	
H18	0.7852	−0.0419	0.1193	0.078*	
C19	0.73596 (8)	0.0858 (5)	0.16203 (18)	0.0770 (10)	
H19	0.7217	−0.0133	0.1775	0.092*	
C20	0.71982 (8)	0.2485 (5)	0.17348 (18)	0.0729 (10)	
H20	0.6945	0.2596	0.1968	0.088*	
C21	0.74032 (9)	0.3942 (5)	0.15128 (18)	0.0774 (10)	
H21	0.7291	0.5045	0.1596	0.093*	
C22	0.77795 (8)	0.3792 (4)	0.11624 (16)	0.0650 (8)	
H22	0.7920	0.4789	0.1007	0.078*	
Cl1	1.197500 (19)	0.25261 (10)	0.01853 (5)	0.0708 (3)	
Cl2	1.04355 (2)	0.33930 (11)	−0.07912 (4)	0.0718 (3)	
N1	0.94269 (5)	0.1515 (3)	0.06772 (11)	0.0413 (5)	
H1	0.9550 (7)	0.163 (3)	0.0265 (14)	0.050*	
N2	0.83373 (5)	0.2042 (3)	0.06907 (11)	0.0428 (5)	
N3	0.85896 (7)	−0.0374 (3)	0.22814 (13)	0.0672 (7)	
O1	1.02216 (4)	0.1736 (3)	0.06353 (9)	0.0548 (5)	
O2	0.94335 (5)	0.1152 (3)	0.19676 (10)	0.0876 (8)	
O3	0.94465 (5)	0.2865 (3)	−0.08352 (10)	0.0602 (5)	
O4	0.88571 (5)	0.3817 (2)	−0.13360 (9)	0.0512 (5)	

### Atomic displacement parameters (Å^2^)

**Table:**

	*U*^11^	*U*^22^	*U*^33^	*U*^12^	*U*^13^	*U*^23^
C1	0.0333 (11)	0.0469 (14)	0.0477 (14)	−0.0048 (11)	0.0088 (9)	−0.0098 (11)
C2	0.0392 (12)	0.0525 (16)	0.0481 (14)	0.0005 (11)	0.0085 (10)	−0.0034 (11)
C3	0.0365 (12)	0.0482 (15)	0.0568 (15)	−0.0009 (11)	0.0050 (10)	−0.0051 (12)
C4	0.0351 (12)	0.0420 (15)	0.0619 (17)	−0.0056 (10)	0.0123 (11)	−0.0102 (12)
C5	0.0456 (14)	0.0446 (15)	0.0509 (15)	−0.0098 (12)	0.0122 (11)	−0.0045 (12)
C6	0.0453 (13)	0.0466 (15)	0.0488 (15)	−0.0050 (11)	0.0058 (11)	−0.0047 (11)
C7	0.0332 (12)	0.088 (2)	0.0467 (15)	−0.0018 (13)	0.0080 (10)	0.0048 (14)
C8	0.0367 (12)	0.0704 (19)	0.0462 (15)	−0.0007 (12)	0.0077 (10)	0.0003 (13)
C9	0.0346 (11)	0.0349 (12)	0.0408 (13)	0.0005 (9)	0.0069 (9)	−0.0042 (10)
C10	0.0379 (12)	0.0401 (14)	0.0417 (13)	−0.0004 (10)	0.0061 (9)	−0.0062 (10)
C11	0.0402 (12)	0.0495 (15)	0.0422 (14)	0.0065 (11)	0.0030 (10)	0.0003 (11)
C12	0.0335 (11)	0.0396 (14)	0.0429 (13)	0.0006 (10)	0.0037 (9)	−0.0053 (10)
C13	0.0387 (12)	0.0448 (15)	0.0503 (15)	−0.0017 (11)	0.0095 (10)	−0.0015 (12)
C14	0.0451 (13)	0.0406 (14)	0.0438 (14)	−0.0024 (11)	0.0071 (10)	−0.0033 (11)
C15	0.0665 (16)	0.0642 (19)	0.0462 (15)	−0.0044 (14)	0.0142 (12)	0.0064 (13)
C16	0.095 (2)	0.0593 (19)	0.0532 (17)	0.0005 (16)	0.0097 (15)	0.0086 (14)
C17	0.0321 (11)	0.0607 (17)	0.0417 (14)	0.0026 (11)	0.0075 (9)	−0.0056 (12)
C18	0.0486 (15)	0.062 (2)	0.083 (2)	−0.0056 (14)	0.0190 (13)	−0.0104 (16)
C19	0.0512 (16)	0.090 (3)	0.089 (2)	−0.0172 (17)	0.0248 (15)	−0.0068 (19)
C20	0.0404 (15)	0.113 (3)	0.065 (2)	0.0102 (17)	0.0119 (13)	−0.0050 (18)
C21	0.0597 (18)	0.076 (2)	0.097 (2)	0.0263 (17)	0.0216 (16)	0.0000 (19)
C22	0.0525 (15)	0.062 (2)	0.081 (2)	0.0180 (14)	0.0167 (14)	0.0102 (15)
Cl1	0.0377 (4)	0.0833 (7)	0.0914 (6)	−0.0088 (3)	0.0170 (3)	0.0030 (4)
Cl2	0.0563 (4)	0.0931 (7)	0.0661 (5)	−0.0070 (4)	−0.0030 (3)	0.0237 (4)
N1	0.0296 (9)	0.0548 (13)	0.0395 (11)	0.0012 (9)	0.0095 (8)	−0.0021 (9)
N2	0.0327 (10)	0.0500 (12)	0.0457 (12)	0.0018 (9)	0.0088 (8)	−0.0022 (9)
N3	0.0656 (14)	0.0708 (17)	0.0651 (15)	−0.0111 (13)	0.0067 (11)	0.0144 (14)
O1	0.0330 (8)	0.0832 (14)	0.0482 (10)	−0.0031 (8)	0.0087 (7)	0.0094 (9)
O2	0.0396 (10)	0.177 (3)	0.0465 (11)	0.0017 (12)	0.0101 (8)	0.0107 (13)
O3	0.0381 (9)	0.0839 (15)	0.0584 (12)	0.0009 (9)	0.0113 (8)	0.0078 (10)
O4	0.0494 (9)	0.0605 (12)	0.0436 (9)	0.0053 (8)	0.0091 (7)	0.0083 (8)

### Geometric parameters (Å, °)

**Table:**

C1—O1	1.363 (2)	C12—N2	1.401 (3)
C1—C2	1.377 (3)	C12—C13	1.415 (3)
C1—C6	1.393 (3)	C13—N3	1.141 (3)
C2—C3	1.384 (3)	C14—O3	1.210 (3)
C2—H2	0.9300	C14—O4	1.334 (3)
C3—C4	1.372 (3)	C15—O4	1.445 (3)
C3—H3	0.9300	C15—C16	1.486 (4)
C4—C5	1.378 (4)	C15—H15A	0.9700
C4—Cl1	1.742 (2)	C15—H15B	0.9700
C5—C6	1.367 (3)	C16—H16A	0.9600
C5—H5	0.9300	C16—H16B	0.9600
C6—Cl2	1.734 (3)	C16—H16C	0.9600
C7—O1	1.415 (2)	C17—C18	1.368 (4)
C7—C8	1.513 (3)	C17—C22	1.370 (3)
C7—H7A	0.9700	C17—N2	1.440 (3)
C7—H7B	0.9700	C18—C19	1.394 (3)
C8—O2	1.213 (3)	C18—H18	0.9300
C8—N1	1.335 (3)	C19—C20	1.365 (4)
C9—C12	1.381 (3)	C19—H19	0.9300
C9—N1	1.395 (3)	C20—C21	1.356 (4)
C9—C10	1.416 (3)	C20—H20	0.9300
C10—C11	1.371 (3)	C21—C22	1.384 (3)
C10—C14	1.458 (3)	C21—H21	0.9300
C11—N2	1.345 (3)	C22—H22	0.9300
C11—H11	0.9300	N1—H1	0.82 (2)
			
O1—C1—C2	124.7 (2)	O3—C14—C10	123.1 (2)
O1—C1—C6	116.3 (2)	O4—C14—C10	112.99 (19)
C2—C1—C6	119.0 (2)	O4—C15—C16	108.3 (2)
C1—C2—C3	120.7 (2)	O4—C15—H15A	110.0
C1—C2—H2	119.7	C16—C15—H15A	110.0
C3—C2—H2	119.7	O4—C15—H15B	110.0
C4—C3—C2	119.1 (2)	C16—C15—H15B	110.0
C4—C3—H3	120.5	H15A—C15—H15B	108.4
C2—C3—H3	120.5	C15—C16—H16A	109.5
C3—C4—C5	121.2 (2)	C15—C16—H16B	109.5
C3—C4—Cl1	119.2 (2)	H16A—C16—H16B	109.5
C5—C4—Cl1	119.60 (19)	C15—C16—H16C	109.5
C6—C5—C4	119.3 (2)	H16A—C16—H16C	109.5
C6—C5—H5	120.3	H16B—C16—H16C	109.5
C4—C5—H5	120.3	C18—C17—C22	120.9 (2)
C5—C6—C1	120.7 (2)	C18—C17—N2	120.8 (2)
C5—C6—Cl2	119.96 (19)	C22—C17—N2	118.3 (2)
C1—C6—Cl2	119.30 (18)	C17—C18—C19	118.9 (3)
O1—C7—C8	109.44 (19)	C17—C18—H18	120.5
O1—C7—H7A	109.8	C19—C18—H18	120.5
C8—C7—H7A	109.8	C20—C19—C18	119.9 (3)
O1—C7—H7B	109.8	C20—C19—H19	120.0
C8—C7—H7B	109.8	C18—C19—H19	120.0
H7A—C7—H7B	108.2	C21—C20—C19	120.7 (3)
O2—C8—N1	123.9 (2)	C21—C20—H20	119.6
O2—C8—C7	118.8 (2)	C19—C20—H20	119.6
N1—C8—C7	117.25 (19)	C20—C21—C22	120.1 (3)
C12—C9—N1	129.6 (2)	C20—C21—H21	119.9
C12—C9—C10	107.26 (19)	C22—C21—H21	119.9
N1—C9—C10	123.14 (18)	C17—C22—C21	119.4 (3)
C11—C10—C9	107.27 (19)	C17—C22—H22	120.3
C11—C10—C14	127.6 (2)	C21—C22—H22	120.3
C9—C10—C14	124.99 (19)	C8—N1—C9	125.75 (19)
N2—C11—C10	109.4 (2)	C8—N1—H1	123.4 (17)
N2—C11—H11	125.3	C9—N1—H1	110.5 (18)
C10—C11—H11	125.3	C11—N2—C12	108.81 (17)
C9—C12—N2	107.22 (19)	C11—N2—C17	125.1 (2)
C9—C12—C13	133.6 (2)	C12—N2—C17	125.30 (19)
N2—C12—C13	118.87 (18)	C1—O1—C7	116.70 (18)
N3—C13—C12	173.9 (2)	C14—O4—C15	116.29 (18)
O3—C14—O4	123.9 (2)		
			
O1—C1—C2—C3	179.2 (2)	C22—C17—C18—C19	0.2 (4)
C6—C1—C2—C3	−0.7 (4)	N2—C17—C18—C19	179.4 (2)
C1—C2—C3—C4	0.2 (4)	C17—C18—C19—C20	−0.2 (5)
C2—C3—C4—C5	0.1 (4)	C18—C19—C20—C21	0.0 (5)
C2—C3—C4—Cl1	178.79 (19)	C19—C20—C21—C22	0.3 (5)
C3—C4—C5—C6	0.2 (4)	C18—C17—C22—C21	0.1 (4)
Cl1—C4—C5—C6	−178.57 (18)	N2—C17—C22—C21	−179.2 (3)
C4—C5—C6—C1	−0.6 (4)	C20—C21—C22—C17	−0.4 (5)
C4—C5—C6—Cl2	179.78 (19)	O2—C8—N1—C9	5.3 (4)
O1—C1—C6—C5	−179.0 (2)	C7—C8—N1—C9	−175.4 (2)
C2—C1—C6—C5	0.9 (4)	C12—C9—N1—C8	−21.0 (4)
O1—C1—C6—Cl2	0.6 (3)	C10—C9—N1—C8	158.0 (2)
C2—C1—C6—Cl2	−179.51 (19)	C10—C11—N2—C12	0.3 (3)
O1—C7—C8—O2	−164.7 (3)	C10—C11—N2—C17	170.5 (2)
O1—C7—C8—N1	15.9 (3)	C9—C12—N2—C11	0.7 (3)
C12—C9—C10—C11	1.5 (3)	C13—C12—N2—C11	−173.6 (2)
N1—C9—C10—C11	−177.7 (2)	C9—C12—N2—C17	−169.5 (2)
C12—C9—C10—C14	−174.7 (2)	C13—C12—N2—C17	16.2 (3)
N1—C9—C10—C14	6.1 (4)	C18—C17—N2—C11	126.0 (3)
C9—C10—C11—N2	−1.1 (3)	C22—C17—N2—C11	−54.7 (3)
C14—C10—C11—N2	174.9 (2)	C18—C17—N2—C12	−65.4 (3)
N1—C9—C12—N2	177.8 (2)	C22—C17—N2—C12	113.9 (3)
C10—C9—C12—N2	−1.3 (2)	C2—C1—O1—C7	6.4 (3)
N1—C9—C12—C13	−9.1 (4)	C6—C1—O1—C7	−173.7 (2)
C10—C9—C12—C13	171.7 (3)	C8—C7—O1—C1	172.4 (2)
C11—C10—C14—O3	−175.9 (2)	O3—C14—O4—C15	0.9 (4)
C9—C10—C14—O3	−0.5 (4)	C10—C14—O4—C15	−177.4 (2)
C11—C10—C14—O4	2.5 (4)	C16—C15—O4—C14	179.6 (2)
C9—C10—C14—O4	177.9 (2)		

### Hydrogen-bond geometry (Å, °)

**Table:**

*D*—H···*A*	*D*—H	H···*A*	*D*···*A*	*D*—H···*A*
N1—H1···O3	0.82 (2)	2.15 (3)	2.813 (3)	137 (2)
C7—H7A···O2^i^	0.97	2.57	3.341 (3)	137
C3—H3···N3^i^	0.93	2.61	3.312 (3)	133

Symmetry codes: (i) −*x*+2, *y*, −*z*+1/2.
